# Arthropod entombment in weathering-formed opal: new horizons for recording life in rocks

**DOI:** 10.1038/s41598-020-67412-9

**Published:** 2020-06-29

**Authors:** Boris Chauviré, Mickal Houadria, Aline Donini, Brian T. Berger, Benjamin Rondeau, Gene Kritsky, Pierre Lhuissier

**Affiliations:** 1 CNRS, IRD, IFSTTAR, ISTerre, Université Grenoble Alpes, Université Savoie Mont Blanc, 38000 Grenoble, France; 2 Biology Centre of Academy of Sciences, Institute of Entomology, Branisovska 31, České Budějovice, Czech Republic; 3 Actias, 44260 Savenay, France; 4 @VelvetBoxSociety, Timberbrook Capital, Philadelphia, PA USA; 5 Laboratoire de Planétologie Et Géodynamique, CNRS UMR 6112, Université de Nantes, BP 92208, 44322 Nantes, France; 6 School of Behavioral and Natural Sciences, Mount St. Joseph University, Cincinnati, OH USA; 7 Univ. Grenoble Alpes, CNRS, Grenoble INP, SIMAP, 38000 Grenoble, France

**Keywords:** Solid Earth sciences, Geology, Mineralogy, Palaeontology

## Abstract

Animal fossils preserved in various geological materials, such as limestone, claystone, or amber, provide detailed information on extinct species that is indispensable for retracing the evolution of terrestrial life. Here, we present the first record of an animal fossil preserved in opal formed by weathering with such high-resolution details that even individual cuticle hairs are observed. The fossil consists of the exoskeleton of a nymphal insect belonging to the order Hemiptera and either the family Tettigarctidae or the Cicadidae. This identification is based on anatomical details such as the tibial and femoral morphology of the forelegs. The exoskeleton of the insect was primarily zeolitized during the alteration of the host rocks and later sealed in opal deposited by silica-rich fluids derived from the continental weathering of the volcanic host rocks. Organic matter is preserved in the form of amorphous carbon. This finding makes opal formed by rocks weathering a new, complementary source of animal fossils, offering new prospects for the search for ancient life in the early history of Earth and possibly other terrestrial planets such as Mars, where weathering-formed opal occurs.

## Introduction

**Figure 1:**
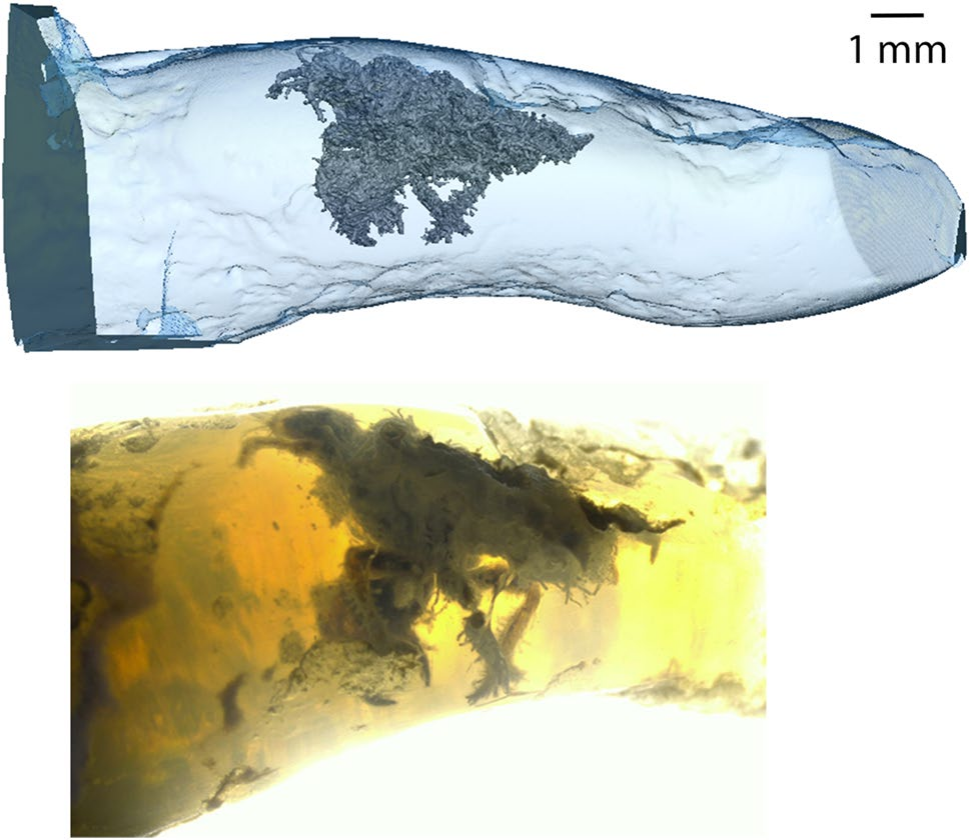
Insect entombed in the opal studied here. 3D surface reconstruction (top) and a microphotograph (bottom). The 3D reconstruction has been performed by X-ray microtomography. The fossil is clearly visible within the near-transparent opal (named “Beverly”). Photo by B. Rondeau and 3D reconstruction by P. Lhuissier.

Most invertebrate fossil records where anatomical details are well preserved are from amber or lacustrine or fluvial sedimentary rocks^1^. These fossils have provided evolutionary evidence of ancestral traits and extinct species, retracing the evolution of life. Here, we describe an insect fossil in opal—a variety of silica—in which the degree of preservation is comparable (Fig. 1)^2^.

Silica minerals exist in various species ranging in structure from amorphous to crystalline. Opal is the main species of amorphous silica that precipitates from saturated or near-saturated silica-rich aqueous fluids produced by the alteration of the earth’s crust. One of the main sources of silica for opal formation (and hence the eventual silicification of fossils) are volcanic rocks, especially volcanic ashes due to their inherent porosity and glass abundance^3–8^.

Among opals, the most sought-after type is precious opal, which differs from common opal only by the presence of colored patches (called play-of-color) that appear to move as the stone is viewed from different angles, an optical phenomenon attributed to the diffraction of visible light by a regular arrangement of silica spheres^9,10^. Both precious and common opal form mainly in two geological settings: (1) hydrothermal alteration occurring when hot water circulates underground and precipitates opal when the temperature drops, e.g., as siliceous sinter (a hard incrustation around hot springs); and (2) continental weathering that occurs when meteoric waters percolate, hydrolyzing silicate while transforming the rock into a soil. In both contexts, the dissolved silica exists as silicic acid (H_4_SiO_2_) in aqueous solution. Silicic acid will polymerize into silica during a variation in physical or chemical conditions, primarily pH and temperature^11^. The degradation of organic matter may favor the precipitation of silica by providing functional groups (hydroxyl groups), making amorphous silica an important preservation agent of fossils^11,12^.

Fossilization involving silica results from permineralization, replacement, or entombment. Permineralization is especially documented for wood and bone when a silica-rich fluid fills the cavities between organ tissues^12–17^. Australian wood and dinosaur bone fossilized in precious opal are an impressive manifestation of permineralization by silica^18,19^. Replacement results from the dissolution of an original organism, creating a mold that can be later filled by the precipitation of silica^7,8,20^. The dissolution or degradation of the original material and precipitation of silica can be concurrent, leading to the exceptional preservation observed in arthropods and brachiopods in the Barstow Formation (California) or the Glass Mountains (Texas)^21–23^. By contrast, entombment is the trapping of an organism in silica that occurs by a rapid silica precipitation on the external surface. This is mainly documented in hydrothermal settings^24–26^, where silica may preserve an entire ecosystem, as observed in the 407-million-year-old Rhynie Chert (Scotland), the oldest fossil terrestrial environment with a comprehensive preservation of flora and fauna^27^. During diagenesis (the hardening of sediments into rocks due to burial), the most amorphous phase of silica (opal-A: amorphous) will transform into more crystalline phases (opal-CT, constituted of nano-domains of cristobalite and tridymite) until it becomes quartz. The transformation of opal into quartz leads to the formation of chert, a sedimentary rock that contains the oldest evidence of life on Earth^28^. Silica entombment associated with hot springs is thus highly studied for its similarity to early Earth environments, and the same process is suspected to have occurred in some locations on Mars^25,26,29^.

Entombment also occurs in the context of continental weathering, where opal develops through the weathering of rocks, mostly of volcanic origin. However, until now, plant roots were the only organisms documented as being preserved through entombment in continental weathering opal in Ethiopia^4,5^. Here, we present novel data on a sample nicknamed “Beverly,” the first occurrence of an animal fossil entombed in gem opal formed by continental weathering. This provides new prospects for life evolution studies by demonstrating that silica entombment can occur during opal formation through the weathering process, which is more widespread than highly localized hydrothermal settings. We discuss the processes that led to the preservation of an insect in opal, and we consider the rarity of such a phenomenon and its implications for science.

## Fossil and host material

### Geological context

Co-author B.T.B. acquired the sample, which came from a precious opal deposit mined in the Genteng Formation, Western Java, Indonesia. The Genteng Formation, dated to the Early Pliocene (5–10 million years old)^30,31^, is located 100 km southwest of Jakarta and covers approximately 1,300 km^2^. This geological unit consists of a series of pumice tuff, tuffaceous sandstone, and claystone deposited from air-fall and/or pyroclastic flow in a terrestrial to littoral environment^31–33^. Each layer has been highly weathered into a paleosoil^33^, and the upper part contains a significant number of silicified wood and other plant remains. The opal-bearing layer is a tuffaceous claystone composed of feldspar, smectite, and volcanic glass associated with zeolite (clinoptilolite)^32^. The presence of zeolite is interpreted by Einfalt^34^ as evidence of limited burial and formation in an open freshwater system, whereas Ansori^32^ proposes a burial diagenesis taking place at a depth of several hundred meters, with temperatures reaching 45 °C. The geochemistry of Javanese opals from the Genteng formation exhibits a signature characteristic of silica produced by continental weathering and leaching of volcanic tuff^32,34^. According to these studies, Javanese opal originates from the weathering of volcanoclastic rocks, especially volcanic glass^32,34^. Large amounts of silica are released to percolating fluids, which precipitate into opal during circulation through porous rocks^32,34^. Ansori^32^ proposed that this silica-rich fluid has been trapped in clay-rich layers, where large cavities may have originated from the degradation of plant remains.

### Material characterization

**Figure 2:**
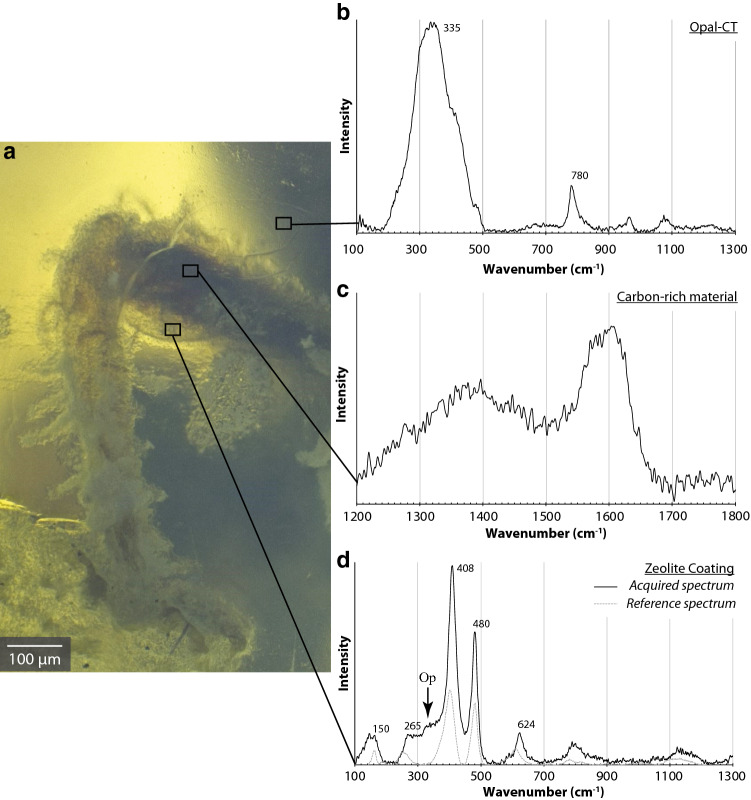
Microphotographs showing the locations of Raman spectra: (**a**) microphotographs of a middle leg of the specimen showing the thin layer coating the fossil. Photo by B. Rondeau. (**b**) Raman spectrum of the matrix with peaks characteristic of opal-CT^35^. (**c**) Raman spectrum of the black carbon-rich material inside the coating. (**d**) Raman spectrum of the thin layer showing features of clinoptilolite–heulandite zeolite. Our Raman spectra are shown in black; in grey—a reference sample^38^. (*Op* opal contribution).

The broad band near 335 cm^−1^ in the Raman spectra (Fig. 2b) of the material that enclosed the fossil clearly indicates opal-CT^35^. The fossil also contains black material that exhibits a weak Raman signal consisting of an asymmetrical broad band near 1,600 cm^−1^, with a weaker band at about 1,360 cm^−1^ (Fig. 2c). These broad bands are typical of G and D bands (respectively) of amorphous carbon^36,37^. A transparent layer 50–100 µm thick coats the surface of the fossil, preserving even the hairs on the cuticle. The Raman spectra of this coating is typical of zeolites from the clinoptilolite–heulandite series^38^: two main bands at 408 and 480 cm^−1^, with additional bands near 150, 265, and 624 cm^−1^ and broad bands near 797 cm^−1^ and 1,130 cm^−1^ (Fig. 2d).

## Taxonomy of the fossil

**Figure 3:**
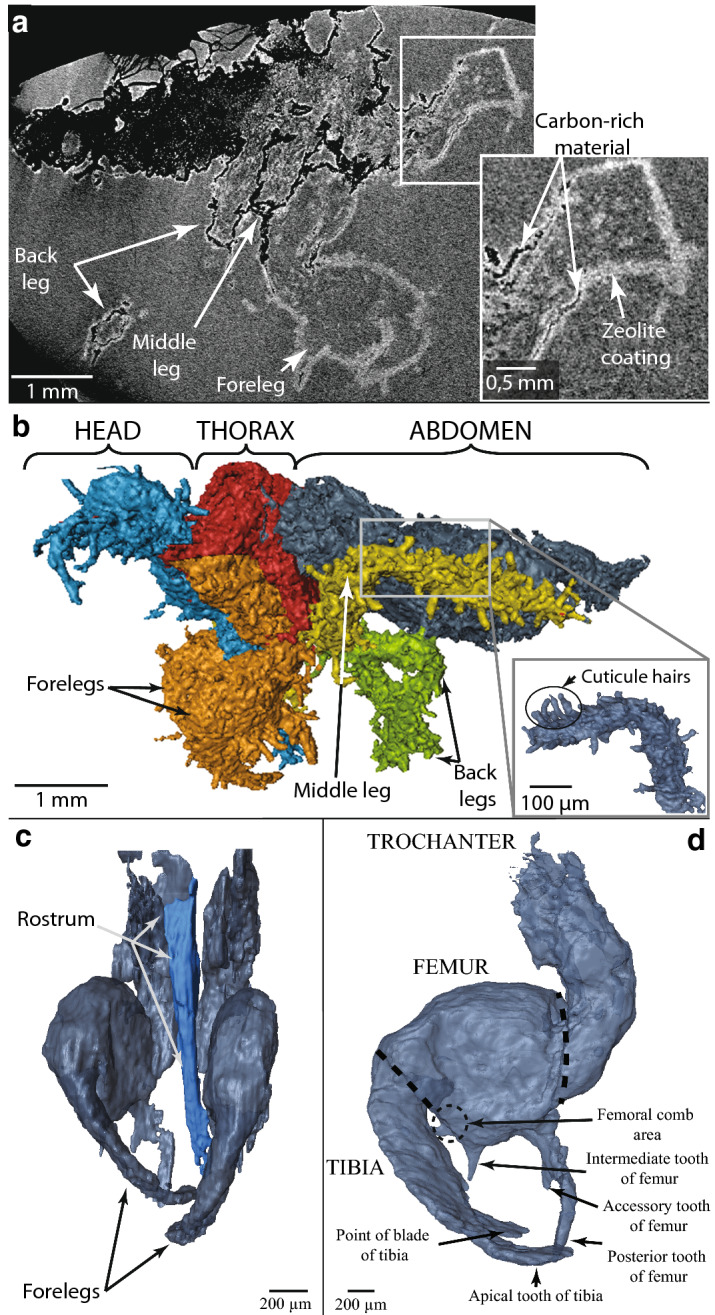
Anatomical details of the fossil. X-ray computed microtomography projections and 3D views. (**a**) X-ray projection of the fossil showing the zeolite coating and the carbon-rich material. This image demonstrates that the material inside and outside the fossil is identical. (**b**) 3D side view of the whole insect exuvia with colored anatomical parts; the inset (bottom right) highlights cuticle hairs. (**c**) Frontal view of the forelegs with rostrum highlighted in blue. (**d**) Internal side view of the right foreleg, after numerically removing the zeolite coating. Animated images corresponding to (**b**), (**c**) and (**d**) are available in Supplementary Information 1.

Taxonomic identification was performed on the basis of scans obtained through non-destructive X-ray computed microtomography (details in “Methods” section). The X-ray computed tomography scans show both the exterior and the interior of the fossil outlined by the coating of lighter zeolite material (Fig. 3a). The interior of the fossil does not exhibit any internal structure and may be composed of the same material as the host (opal).

The 5.5 mm long specimen includes the head, the thorax with three pairs of legs, and part of the abdomen (Fig. 3b) and is undoubtedly from a nymphal insect. Both microphotography (Fig. 2) and high-resolution tomography (Fig. 3b) allowed us to distinguish individual cuticle hairs, highlighting the excellent preservation.

The tomography and 3D reconstruction provide vital information used to identify the insect superfamily. The prominent rostrum (sucking mouthparts; Fig. 3c) identifies the specimen as an insect of the order Hemiptera^39^. To reveal greater detail on the forelegs, the interior of the zeolite coating was reconstructed, and the exterior was numerically removed. This reveals the tibial and femoral morphology of the forelegs (Fig. 3d) and identifies the specimen as a nymph (immature form) of a hairy cicada or a true cicada^40^.

## Discussion

X-ray computed microtomography shows that both the interior and the exterior of the fossil are coated by a thin layer of lighter material, identified as zeolite by Raman spectroscopy (Fig. 3a). No internal structure has been observed and the interior is occupied by the same opal as that outside the fossil (based on Raman spectroscopy and X-ray). These observations indicate that the insect integument was hollow, suggesting that it may be an exuvia (the shed exoskeleton of an insect). An alternative explanation may be that the insect died in the soil, and the fluids dissolved the soft interior parts of the insect. The more resistant cuticle would have been preserved and subsequently fossilized.

Morphological details (especially the rostrum and the prominent forelegs) identify the fossil as a cicada nymph belonging to either the Tettigarctidae (known as hairy cicadas) or the Cicadidae (otherwise called true cicadas) families. According to the existing literature, fossils of immature cicadas are rare. To date, only four have been discovered, all in amber. Of these cicada fossils, two are new records: one in the Dominican Republic (*Dominicicada youngi*) and the other in Myanmar (*Burmacicada protera*)^41^. A third cicada specimen was found in Baltic amber, but no taxonomic description has been published so far^42^. Fragments of another nymph have also been reported in Cretaceous amber from New Jersey^43^.

Nymphs of the two families can only be distinguished metamorphically using features such as the number of femoral spines or the presence or absence of the tarsi, which only develop during the last nymphal stage (instar)^44^. The size of the nymph (5 mm) and the shape of the slightly swollen zeolite layer on the femoral comb (Fig. 3d) lead us to believe that it is an early instar^44^, rendering identification to one of the families impossible. In terms of phylogeographical context, the presence of the two families on both sides of the Wallace line^45–50^ (a faunal boundary separating two different ecozones in southeast Asia) prevents any clear assertion of this specimen belonging to the Cicadidae rather than the Tettigarctidae.

The amorphous carbon composing the black, carbon-rich material (according to the Raman signature^36,37^) could result from the transformation of organic material composing the fossil. This material is coated by clinoptilolite–heulandite zeolites (Fig. 2). The zeolitic coating is consistent with the clinoptilolite identified by Einfalt^34^ using X-ray diffraction. As zeolites are observed exclusively at the interface between the organic material and the host opal, we propose that zeolitization occurred before the precipitation of opal. Therefore, opal precipitation may not have occurred immediately or concurrently after the zeolitization, although opal precipitation ultimately preserves the fossil.

Clinoptilolite–heulandite are the most common zeolitic series found in sedimentary and igneous rocks; they form in various environments, including hydrological open and closed systems, deep marine sediments, soils, through hydrothermal alteration, and during diagenesis and burial metamorphism, especially from volcanic material^38,51,52^. The presence of clinoptilolite–heulandite zeolites has been discussed by Einfalt^34^ and Ansori^32^, both concluding that they originate from the destabilization of volcanic glass contained in the opal-bearing or overlying layers. However, the conditions of formation proposed by each author differs; Einfalt^34^ proposed that no burial diagenesis occurred, in contradiction to Ansori^32^. The fine details preserved in our sample could be an evidence of a limited burial, as higher pressure might have obliterated them. Zeolitization may have played a significant role in preventing alteration of the fossil in the aqueous environment required for opal precipitation. Unfortunately, the zeolite coating the insect cuticle partially hides the morphological details required for a more thorough taxonomic identification.

**Figure 4:**
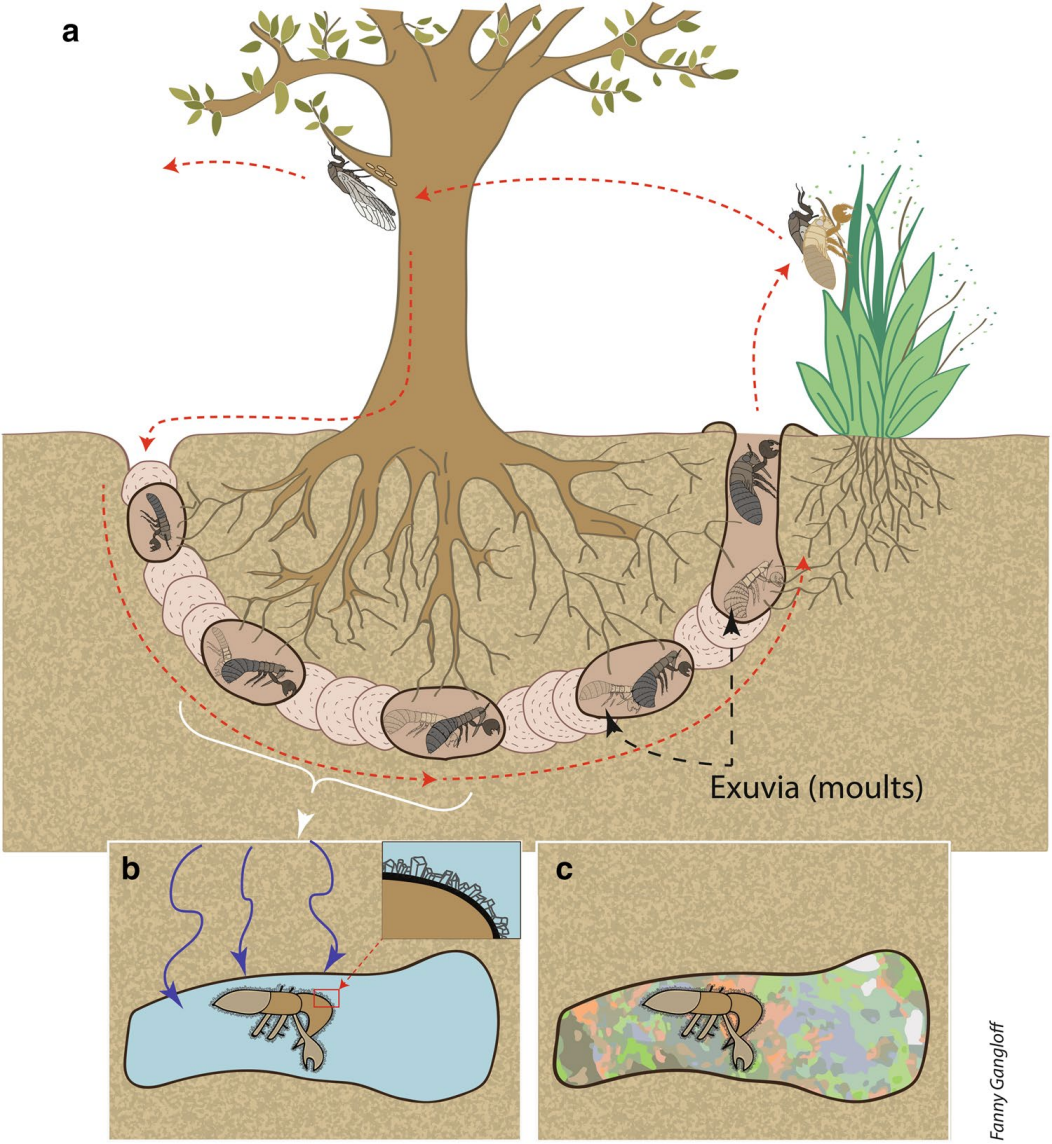
Scenario of sample formation. (**a**) Life cycle of an extant cicada. The white bracket identifies the instars corresponding to our sample compare with Fig. 3. (**b**) Fossilization of cicada exuvia. Percolating fluid (represented by blue arrows) fills the cavity, and zeolites crystallize on exuvia. (**c**) The precipitation of opal sealed the exuvia in place, forming the well-preserved fossil. Drawing by F. Gangloff.

The identification of the fossil as a true or hairy cicadas provides information on the genesis of our sample. Indeed, in extant species of cicada, after eggs laid in branches have hatched, the first instar nymphs fall to the ground^53,54^ (Fig. 4). They burrow into the soil with their strong front legs, using the other two pairs of legs to move through a narrow burrow^54^, searching for roots from which they suck xylem fluid. The many cuticle hairs observed on our specimen (Fig. 2b) play an important role in aggregating loosened earth behind the tunneling nymph. Cicada nymphs are usually found between 5 and 80 cm deep in the soil, but they have been reported from up to 1.2 m below ground^54,55^. The nymphs feed on tree roots, usually going through four instars underground before the final instar nymph emerges from the soil to transform into an adult^53^. The time required for this subterranean development varies among different species from 1 to 17 years^56^.

Opal is a common product of the weathering of volcanic glass^57,58^ and precipitates from an aqueous gel^9^. Opal precipitation could originate from the weathering of volcanic rocks deposited later, rather than occurring simultaneously with zeolite crystallization, as demonstrated for both precious opal formation^3–6^ and fossils replaced by silica in various location^7,8,20^.

Because cicada nymphs live in shallow soils^53–55^ (Fig. 4) and paleosoils have been documented in the Genteng formation^31–33^, we consider that a shallow soil is the most probable environment for the genesis of our sample. Moreover, the excellent preservation of the fossil, particularly the conservation of cuticle hairs (Fig. 3b), is inconsistent with exhumation and transport that would have damaged or even obliterated these delicate features. We therefore propose that the process of zeolitization occurred shortly after the shedding of the nymphal skin. This first stage of zeolitization has been observed in plant fossils preserved in Ethiopian opals^4–6^.

The encrustation of insects in opal is a rare phenomenon. The few previously documented examples have originated in hydrothermal settings, where silicification rapidly entombs organisms^24–27,59^. Opal formation through continental weathering is a much more widespread phenomenon, but is also slower. Animals such as insects would normally crawl away before the process could begin—in contrast to fossilization in amber, where insects are literally glued into the sap that forms the amber. In addition, insect cuticles are usually hydrophobic, which tends to isolate the insect from silica-rich water^60^. Only immobile, attached invertebrates (dead or alive) may be entrapped by the silica-rich fluids, which gradually turn into a gel and finally into opal. Hence, it is much more probable that plants rather than animals would be preserved as fossils in opal. This supports the idea that this fossil is of a lifeless, immobile cicada exuvia. Specific geological and environmental conditions were necessary to preserve our sample and a detailed geological investigation of the opal mine is required to determine these conditions more fully.

## Implications

In this study, we demonstrate that opal forming through continental weathering can preserve insects and traces of organic compounds nearly as efficiently as hydrothermal siliceous sinters or amber. We also demonstrate that X-ray computed tomography is an efficient technique to detect and characterize fossils preserved in opal. Even if opals may be opaque, making visual examination of the interior impossible, tomography could still be used to reveal entombed specimens and hence uncover new paleontological or evolutionary clues of ancient life. Opal precipitates from fluids enriched in silica through the weathering of silicate crust, a process that has operated since the Archean times. Preservation in weathering-formed opal is therefore a potential source of information about the evolution of life in the earliest periods of Earth’s history. Moreover, preservation in amber overrepresents insect fossils from forest habitats, due to its formation from tree resin, and insect fossils in sedimentary rocks mainly sample communities from aqueous environments^61^. The rarity of cicada nymphs compared to other insects^62^ could be related to their underground habitat, where fossilization is scarcer. Therefore, weathering-formed opal here demonstrates its potential for a better understanding of the evolution of species that live underground. Opal’s capacity to preserve fossils provides new opportunities in the search for ancient and/or extraterrestrial life. Amorphous silica is present on the surface of Mars^29,63,64^, and is already targeted as the most promising source for the search for extraterrestrial life. The fossil-preserving potential of amorphous silica has been mostly observed in samples that formed in hydrothermal environments^25,26,65^, whereas opaline silica deposits have been identified as originating from the weathering of its silicate crust^66,67^. The future landing site of NASA’s next rover missions on Mars (Mars 2020) includes opaline silica that is thought to be of weathering origin^68^. Even if opaline silica is already the most encouraging mineral for exploring the traces of extraterrestrial life, its potential is now widened to a larger range of geological settings, thus offering a new possibilities for a record of life forms on Mars.

## Methods

### Microphotography

Microphotographs have been acquired using a Keyence VHX-2000 numerical microscope allowing Z-stack to enhance field depth. We used a VH-Z20T zoom lens allowing magnification from × 20 to × 200, operated in transmitted light without polarizing filters. To improve the exposition of some parts, additional light has been added by external optical fibers.

### X-ray microtomography

3D images have been acquired by x-ray microtomography on RX-Solutions EasyTom XL Nano equipment using a Hamamatsu L8121 sealed tube and a Paxscan 2520DX flat panel from VAREX Imaging. A source tension of 100 kV with a current of 80 µA was combined with a 0.25 mm thick aluminum filter. The magnification was set to 25.4 resulting in a voxel size of 5 µm. 1984 projections were acquired over one turn. Each project is the average of 6 images exposed during 0.5 s each. A classical filtered back projection algorithm was used for 3D reconstruction.

In order to reveal fine details on forelegs, the reconstruction has been segmented slice by slice by manual border selection using the open software ImageJ. 3D renderings are obtained with the ThermoFicher Scientific Avizo software.

### Raman spectroscopy

Raman spectra were acquired using a Labram Jobin–Yvon Raman spectrometer (2 cm^–1^ resolution) equipped with an Ar laser emitting at 514 nm, 50 mW power, combined with a microscope with a 50 × objective.

## Supplementary information

Supplementary file1 (PDF 48783 kb)
