# Supplementary material for: Arthropod entombment in weathering-formed opal: new horizons for recording life in rocks
Source: Sci Rep. 2020 Jun 29;10:10575. doi: 10.1038/s41598-020-67412-9 (PMC7324577; doi:10.1038/s41598-020-67412-9)

Arthropod entombment in precious opal: new horizons for recording life in rocks

SUPPLEMENTARY MATERIALS

Chauviré Boris*

Univ. Grenoble Alpes, Univ. Savoie Mont Blanc, CNRS, IRD, IFSTTAR, ISTerre, 38000 Grenoble, France

Houadria Mickal

Institute of Entomology, Biology Centre of Academy of Sciences, Branisovska 31,c, Czech Republic

Donini Aline

Actias, 44260 Savenay, France

Berger Brian T.

@VelvetBoxSociety, Timberbrook Capital, Philadelphia PA, USA

Rondeau Benjamin

Université de Nantes, Laboratoire de Planétologie et Géodynamique, CNRS UMR 6112, BP 92208, 44322 Nantes, France

Kritsky Gene

School of Behavioral and Natural Sciences, Mount St. Joseph University, Cincinnati, Ohio, USA

Lhuissier Pierre

Univ. Grenoble Alpes, CNRS, Grenoble INP, SIMAP, F-38000 Grenoble, France

Movie S1: Animated images of global fossil with colored parts.

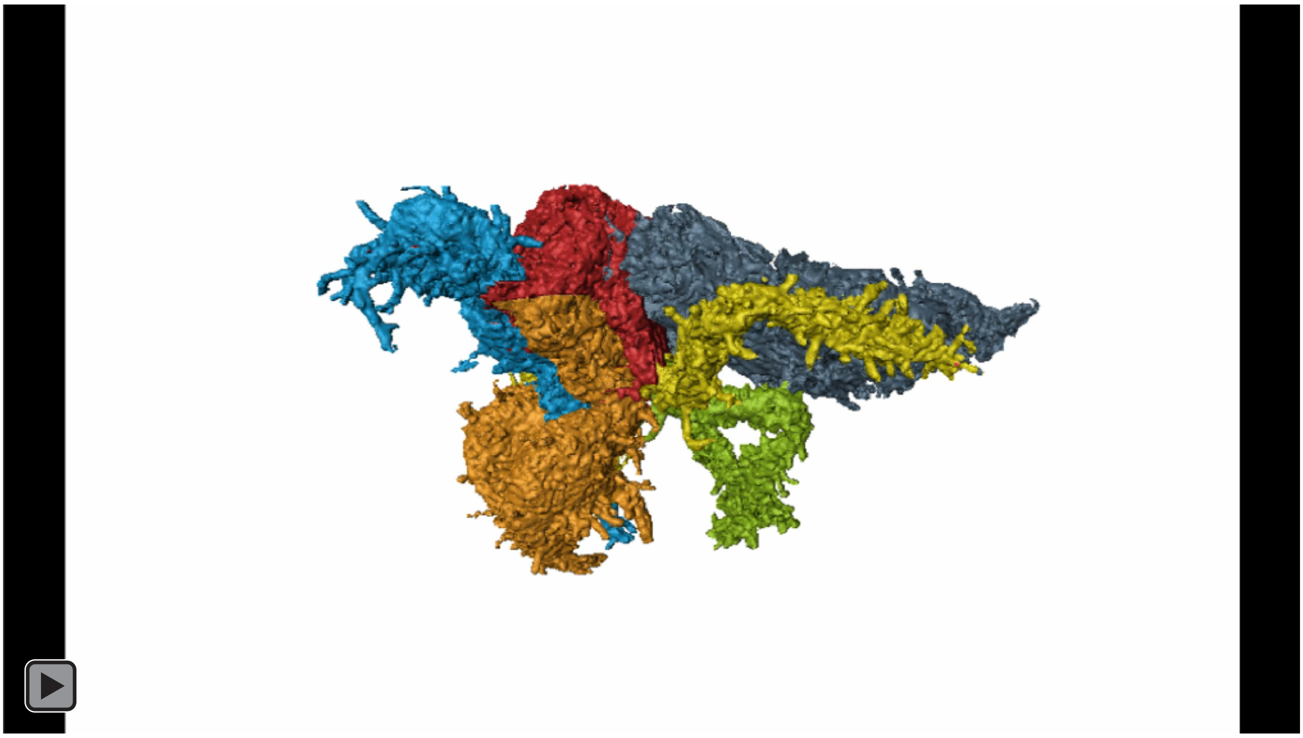

Movie S2: Animated images of forelegs and rostrum.

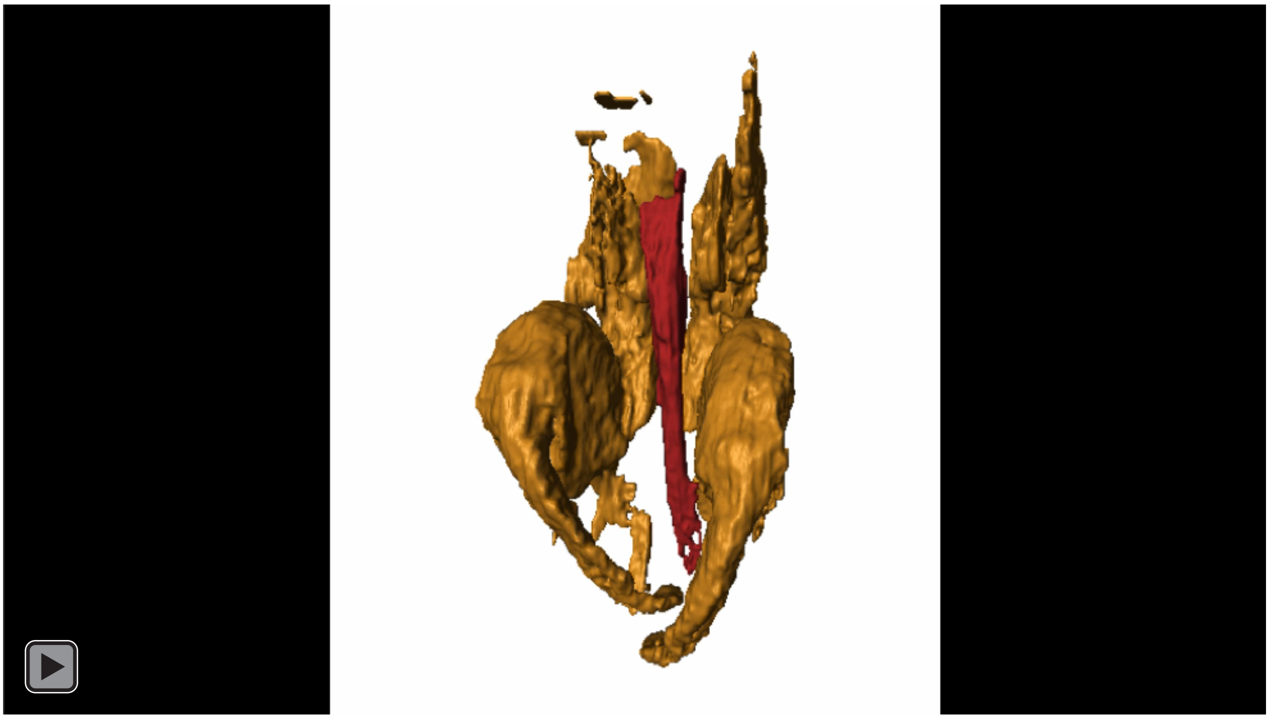

Movie S3: Animated images of the right forelegs.

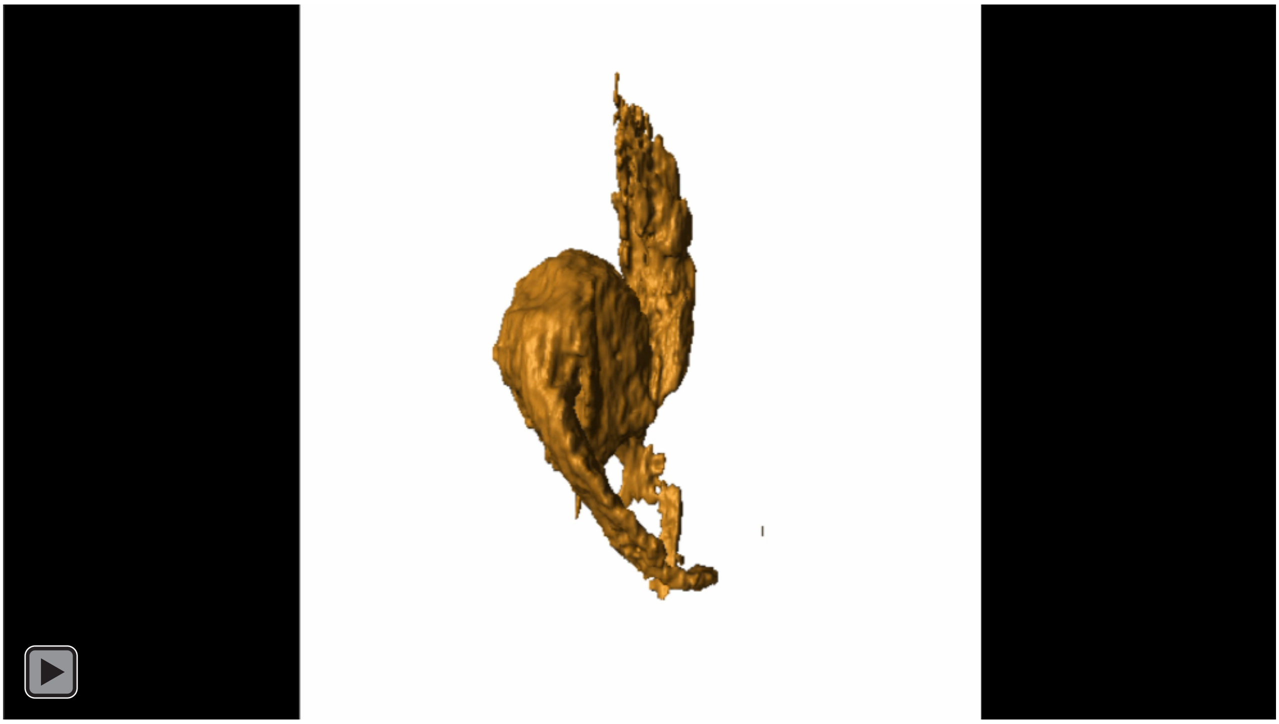

Supplement: Supplementary file 1 — Supplementary file1 (PDF 48783 kb) [file 41598_2020_67412_MOESM1_ESM.pdf]
